# A Novel Method of Synthesizing Graphene for Electronic Device Applications

**DOI:** 10.3390/ma11071120

**Published:** 2018-06-30

**Authors:** Nierlly Galvão, Getúlio Vasconcelos, Rodrigo Pessoa, João Machado, Marciel Guerino, Mariana Fraga, Bruno Rodrigues, Julien Camus, Abdou Djouadi, Homero Maciel

**Affiliations:** 1 Centro de Ciência e Tecnologia de Plasmas e Materiais—PlasMat, Instituto Tecnológico de Aeronáutica, 12228-900 São José dos Campos, SP, Brazil; marcielguerino@yahoo.com.br (M.G.); bruno.manzolli@gmail.com (B.R.); homero@ita.br (H.M.); 2 Photonics Division, Instituto de Estudos Avançados, Rodovia dos Tamoios, 12228-001 São Jose dos Campos, SP, Brazil; getulio@ieav.cta.br; 3 Universidade Brasil, Rua Carolina Fonseca 235, 08230-030 São Paulo, SP, Brazil; mafraga07@hotmail.com; 4 Associate Laboratory of Sensors and Materials, Instituto Nacional de Pesquisas Espaciais, 12227-010, São José dos Campos, SP, Brazil; machadopaulo@gmail.com; 5 Institut des Matériaux Jean Rouxel IMN, UMR 6502, Université de Nantes, 2 rue de La Houssinière, BP 32229, Nantes Cedex 44322, France; julien.camus@cnrs-imn.fr (J.C.); abdou.djouadi@cnrs-imn.fr (A.D.)

**Keywords:** graphene synthesis, silicon carbide, thin film, high-power impulse magnetron sputtering, thermal decomposition, electronic devices

## Abstract

This article reports a novel and efficient method to synthesize graphene using a thermal decomposition process. In this method, silicon carbide (SiC) thin films grown on Si(100) wafers with an AlN buffer layer were used as substrates. CO_2_ laser beam heating, without vacuum or controlled atmosphere, was applied for SiC thermal decomposition. The physical, chemical, morphological, and electrical properties of the laser-produced graphene were investigated for different laser energy densities. The results demonstrate that graphene was produced in the form of small islands with quality, density, and properties depending on the applied laser energy density. Furthermore, the produced graphene exhibited a sheet resistance characteristic similar to graphene grown on mono-crystalline SiC wafers, which indicates its potential for electronic device applications.

## 1. Introduction

Nowadays, the synthesis of high-quality graphene has been the focus of several researchers due to the great potential applications of this material in electronic devices, sensors and flexible displays [1].

Among the graphene synthesis methods, the thermal decomposition processes have been successfully used to grow graphene layers on silicon carbide (SiC) [2,3,4,5]. In general, these studies show the use of SiC wafers as substrates to be decomposed by heating, using an induction furnace at vacuum or at atmospheric pressure with an inert gas flow [6,7,8,9,10]. The kinetics of graphene formation and properties, such as structure and morphology, have been shown to be dependent on the reactor pressure, type of gas atmosphere, orientation, and face termination of the SiC wafer [10,11,12].

Most recently, the use of a laser beam as a heating source for graphene formation from SiC has been reported [13,14,15]. The focus of these studies was to investigate the growth of graphene on the Si- and/or C-face terminations of SiC wafers. Perrone et al. used a near infrared Nd:YVO_4_ (1064 nm) laser to promote the heating of the SiC surface [13]. They reported the possible presence of graphene when this process was performed using argon gas flow, or in a vacuum at a pressure of 10^−5^ Torr. Using an ultra-high vacuum (UHV), only a disordered graphite phase was observed [13]. Lee et al., using an UV laser (248 nm), noticed not only that it is possible to grow epitaxial graphene (EG) from Si-terminated SiC (0001), but also pointed out that EG has a structure comparable to thermally-grown graphene under UHV using the same substrate [14]. Unlike the previous two studies, Yannopoulos et al. obtained graphene on SiC without the use of a vacuum environment or pre-treatment of the SiC substrate [15]. In their work, a carbon dioxide (CO_2_) laser beam was used as the heating source, and the argon gas flow at atmospheric pressure was applied to form layers of EG. An advantage of using a CO_2_ laser is the cooling effect during pulsing, and the possibility of writing graphene patterns on SiC, which eliminates the lithographic step [15]. Nevertheless, the aforementioned studies and their processes have a serious drawback—the use of high-cost SiC wafers as a substrate [16,17].

In another recent study, Galvão et al. reported on the growth of graphene layers on a low cost polycrystalline SiC substrate obtained from powder metallurgy using a CO_2_ laser beam [18]. Although the graphene obtained may be applied in several areas, this SiC substrate is not ideal for microelectronic applications. In order to improve the quality of the graphene samples without a significant increase in production costs, we explored the use of SiC thin films as substrates in combination with the CO_2_ laser beam heating technique for EG growth. Herein, SiC thin films were grown by high-power impulse magnetron sputtering (HiPIMS) on Si substrates covered with an aluminum nitride (AlN) buffer layer. To the best of our knowledge, the formation of graphene from SiC thin films grown on AlN/Si substrates using a CO_2_ laser beam has not yet been reported in the literature [19]. The growth of crystalline SiC films directly onto silicon substrates is known to be difficult due to the large thermal stress between these materials. The literature shows that extensive effort has been devoted to reducing this problem, and that using the AlN buffer layer is a good choice [20,21,22]. The role of the AlN buffer layer is to allow the growth of a high-quality SiC film with a lower density of defects. Subsequently, high-quality graphene can be obtained by thermal decomposition of the SiC film. The growth of graphene on AlN buffer could be important in the development of new electronic device applications, such as high frequency resonators [23]. Furthermore, there is a growing interest in the integration of graphene into wide bandgap semiconductors for various applications [24].

In this work, different levels of laser energy density were applied during SiC thermal decomposition, and the chemical properties and quality of the graphene were evaluated using Raman spectroscopy. Moreover, to further understand the material characteristics, morphological and electrical measurements were performed using atomic force microscopy (AFM) and the four points probe method, respectively.

## 2. Materials and Methods 

### 2.1. SiC Thin Film Growth

SiC thin films were deposited by HiPIMS on pieces of polished p-type Si(100) wafer covered with AlN buffer in a high-vacuum chamber with a background pressure of 6 × 10^−6^ Torr. More details of the magnetron sputtering system can be found elsewhere [25,26]. The working pressure of the argon gas (99.999%) was maintained at 3 × 10^−3^ Torr for a corresponding flow rate of 20 sccm. A high-purity SiC (99.5%, Kurt J. Lesker Company, Jefferson Hills, PA, USA) target with a 4 inch diameter was used. The sputtering reactor was powered by a HiPIMS power supply (Solvix HIP^3^ 5 kW) with an applied power of 200 W and duty cycle of 5%. The target–substrate distance was approximately 65 mm and the deposition was performed for 10 min. The substrate holder was in a floating potential. Before starting the SiC deposition, a pre-sputtering period (10 min at 200 W) was performed to remove contamination from the target surface. The obtained SiC film on the AlN/Si substrate had an average thickness of 240 nm.

The AlN buffer layer was grown on the Si(100) substrate using HiPIMS at Institut des Matériaux Jean Rouxel in Nantes University. More details can be found elsewhere [27,28]. The AlN film on the Si substrate had an average thickness of 1300 nm, and the main crystallographic orientation was (002).

### 2.2. SiC Sublimation by CO_2_ Laser Heating

Heating of the SiC thin film was carried out using a CO_2_ laser (Synrad Evolution—125) with a beam diameter of 200 µm, which emits infrared laser radiation at a wavelength band of 10.6 µm. The samples were positioned 5 mm below the laser focal point (see schematic diagram of the laser irradiation process in [18]). For all processes, a beam overlap of 50% was used. Three different laser scanning velocity rates were used: 2300, 2500 and 2600 mm s^−1^. For all conditions, the laser power applied was 50 W, which corresponded to 40% of the overall power. The entire process occurred under ambient atmosphere and pressure conditions. The energy densities of each scanning velocity were calculated and are shown in Table 1. In this investigation, the energy densities were between 127–145 J cm^−2^, whereas in previous studies, the values were between 132–200 J cm^−2^ [18]. A lower energy density range was chosen because the properties of the synthesized graphene at higher laser energy densities did not present any significant difference in comparison with samples produced with lower energy densities [18].

### 2.3. Material Characterization

The structure of the as-deposited SiC films was investigated using Grazing Incidence X-ray Diffraction (GIXRD) with an incidence angle (ω) of 0.3° operated with a PANalytical X’pert Pro x-ray diffractometer (Almelo, the Netherlands) with CuKα radiation.

Raman analyses were performed using a Horiba Raman microprobe system (LabRAM HR Evolution, Horiba, Kyoto, Japan) equipped with an argon ion laser (514.5 nm). Raman spectra of the SiC and graphene samples were obtained at room temperature in the range of 200–1100 cm^−1^ and 1200–2900 cm^−1^, respectively. The Raman spectral imaging (or Raman mapping) were obtained by focusing on the main characteristic bands (or peaks) of graphene. The type of defect present in the graphene film was inferred according to Eckmann et al. [29], which reported on a relationship between the intensity (height) of the ID and ID’ peaks with the type of defect present in the graphene sample. The maximum ID/ID’ ratio (~13) would correspond to sp^3^-defects, (~7) for vacancy-like defects and, (~3.5) for boundary defects [29]. In this work, the D’ peak is merged with the G peak. To determine the intensity of the D’ peak, a Lorentzian double peaks function fitting was applied to each Raman spectrum.

The surface morphology was investigated by atomic force microscopy (Veeco Instruments Inc, Plainview, NY, USA) (AFM Veeco Multimode with a Nanoscope V control station). The tapping mode was used for all samples except for sample C2, where contact mode was applied because it provided images with better resolutions.

## 3. Results and Discussion

### 3.1. SiC Thin Film Structure

**Figure 1:**
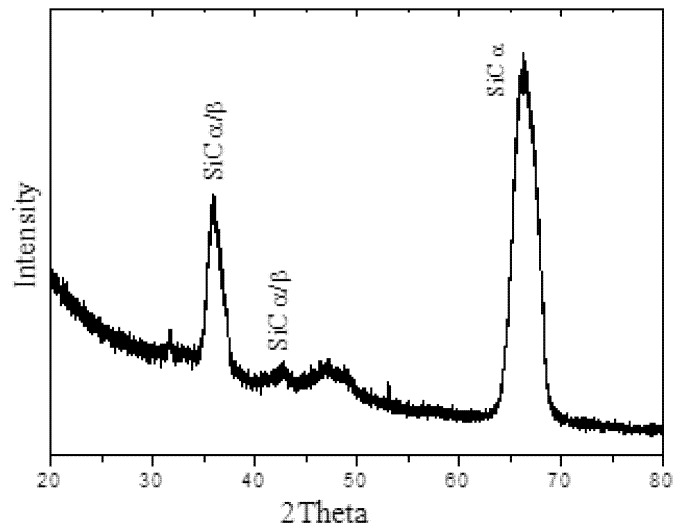
Grazing incidence x-ray diffraction spectrum of SiC film grown on AlN/Si(100) substrate.

In the literature, there is only one report of SiC growth on a Si substrate using HiPIMS [30]. However, due to the large lattice mismatch between SiC and Si (~20%), the grown film exhibited amorphous characteristics and a high residual stress [28,30]. To reduce these effects, several studies have demonstrated that using a sacrificial layer (buffer layer) on the Si substrate before the deposition of SiC is an effective alternative. In this work, we chose to use AlN buffer, which presents a mismatching in the lattice constant of less than 1% compared to SiC. Figure 1 shows the GIXRD spectrum of the SiC thin film grown on AlN/Si(100) substrate. As expected, the identified peaks reveal the polycrystalline nature of the SiC.

In addition, the orientations of SiC indicated in the GIXRD spectrum agree with some studies in the literature [31,32,33]. We found that the peaks of the SiC matched well with those of *α*-SiC (6*H*-SiC), however, it can be not excluded that *β*-SiC (3*C*-SiC) exists.

### 3.2. Graphene Characterization

Raman spectroscopy is a very useful technique to obtain important information about inherent features of carbon materials, such as graphene [34,35,36,37]. The Raman spectra of graphene contains three main in-plane vibrational bands: (i) G-band (~1584 cm^−1^), corresponding to the doubly degenerate E_2g_ phonon mode at the Brillouin zone center; (ii) D-band (1200–1400 cm^−1^), that arises from TO phonons around the K point and requires a defect for its activation; and (iii) 2D-band (2400–2800 cm^−1^), which is the second order of the D-band and has been widely used to evaluate the number of layers and structural quality of graphene [34,37].

**Figure 2:**
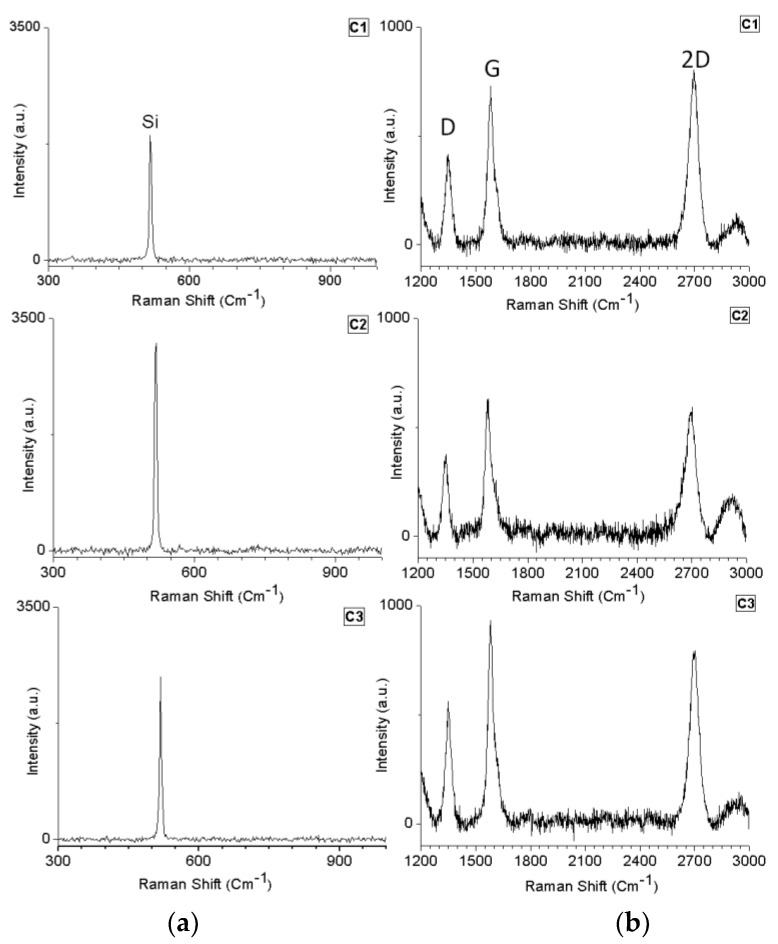
Raman spectra of the samples C1, C2 and C3: (**a**) scanning in the SiC area (column A); (**b**) scanning in the graphene area (column B).

**Figure 3:**
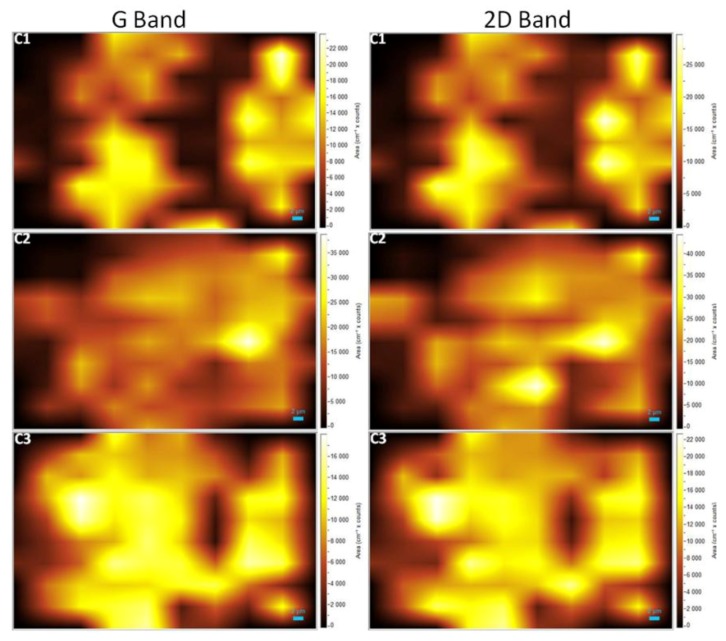
Raman mapping of the samples corresponding to G and 2D bands.

**Figure 4:**
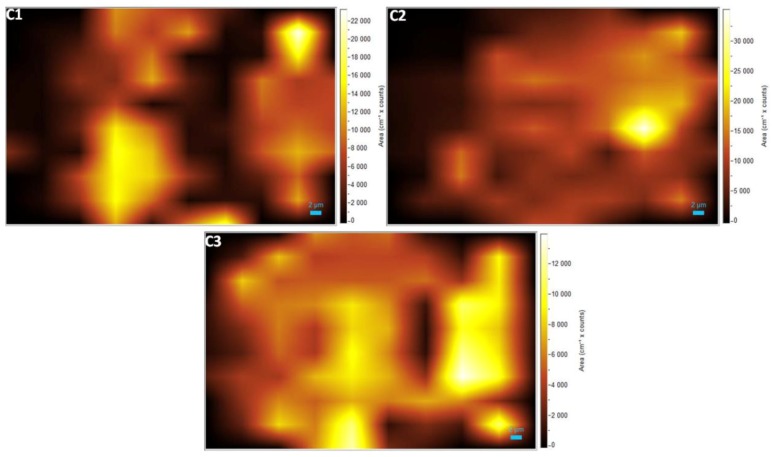
Raman mapping of the samples corresponding to D band.

Figure 2 shows the Raman spectra of each SiC sample that was laser treated. In addition, the Raman spectra of the unexposed SiC area are also presented. The presence of graphene G and 2D-bands and a significant number of defects (D-band) was observed (Figure 2b). Conversely, we noticed the absence of peaks related to the Si–C band (Figure 2a). The absence of the Si–C bands can be an indication that, at this point, all the SiC was decomposed to form graphene. To verify the growth behavior and distribution of graphene in the treated sample, Raman mapping was performed (Figure 3 and Figure 4).

The bright spots observed in the Raman maps (Figure 3 and Figure 4), which are due to the presence of bands related to graphene, agree with the Raman spectra shown in Figure 2. The dark areas correspond to the absence of bands related to graphene. The mapping images also reveal that the graphene did not expand enough to cover the entire region of analysis, forming some “graphene islands”. Furthermore, it is possible to observe a non-uniform distribution of higher intensity regions. This variation is probably related to the quality of the graphene. Several studies reported that the kinetics of graphene growth on the Si- or C-face of SiC is distinct [3,36,37,38,39,40]. When EG is grown on the Si-face under UHV, the rate of sublimation is reduced, and therefore it is possible to control the growth of the graphene layers. This leads to the formation of a large and homogeneous monolayer [41,42].

**Figure 5:**
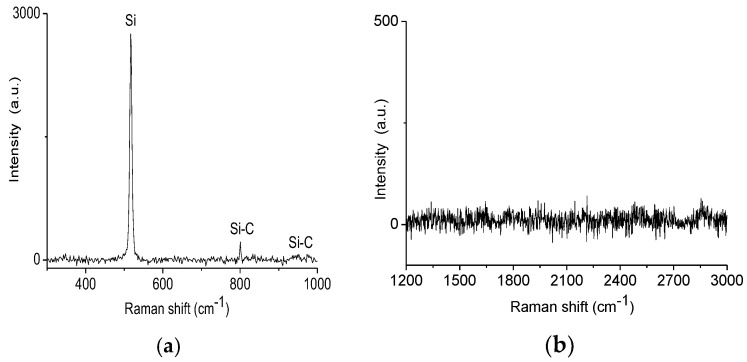
Raman spectra of the dark areas for C2 sample: (**a**) scanning in the SiC range (column A); (**b**) scanning in the graphene range (column B).

When the C-face is considered, the growth rate is higher and its control is more difficult, which usually results in the formation of an inhomogeneous graphene layer [3,38,39]. However, Hass et al. reported that graphene grown on the C-face of the SiC substrate in a RF furnace can be of exceptional quality [43]. This indicates that depending on the technique, it is possible to obtain high quality graphene on both faces. In our studies, graphene was grown on SiC thin film with undefined face-termination (Si–C face). Currently, there is a clear lack of studies reporting on the kinetics of graphene growth on SiC thin films using thermal decomposition by CO_2_ laser heating. Thus, according to the behavior observed by Raman mapping, and considering that the SiC film is polycrystalline and contains some amorphous areas, it is possible to presume that graphene was grown on both Si–C face terminations. Growth on these two possible faces can result in graphene regions with a high defect concentration and different layers. In addition, the laser heating may have also influenced the growth and quality of the graphene. As obtained by Galvão et al. [18], the limited growth of graphene and non-dissociation of SiC in some regions of the material may have been influenced by the heat transfer along and across the heterogeneous surface. Inhomogeneities on the surface cause a non-uniform temperature distribution. A detailed Raman analysis performed on sample C2 revealed that the dark areas on the maps are composed of SiC films that did not get enough energy to dissociate and form graphene; however, crystallization started to take place instead, which can be verified by the well-defined SiC peak showed in Figure 5.

Finally, regarding the quality of graphene, the type of defects present in the sample were determined from the ID/ID’ that was calculated from the Raman spectrum, collected at five random points of each sample. The values of the ID/ID’ found were 3.7 ± 1.0, 4.1 ± 0.5 and 3.7 ± 0.7 for the samples C1, C2 and C3 respectively. According to Reference [18], these results indicate the presence of boundary defects.

### 3.3. Sheet Resistance and Morphology of SiC Thin Film and Graphene Layers

As graphene is a material of great interest for electronic device applications, we have also analyzed the sheet resistance for each sample produced by the developed method (Table 2). In all conditions, the sheet resistance is lower than the sheet resistance of SiC, but only the C2 sample presented properties consistent with graphene. The high sheet resistance presented by C1 and C3 may have occurred because the islands of graphene produced are small and not interconnected. Both Raman maps and AFM surface morphology images endorse this fact.

**Figure 6:**
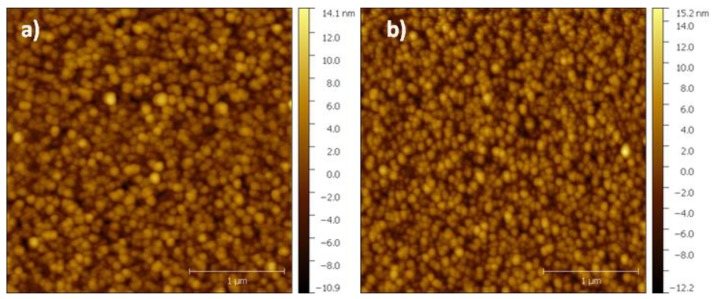
Atomic force microscopy images of SiC (**a**) and AlN (**b**) films.

Before analyzing the surface morphology of the treated samples, it was important to verify the morphology of the SiC surface. Figure 6 shows the AFM images of the surface of the SiC films and the AlN buffer layer before laser treatment. As shown, the SiC film follows the morphology of the AlN layer with grains less than 100 nm. These results indicate high quality SiC and AlN thin films.

**Figure 7:**
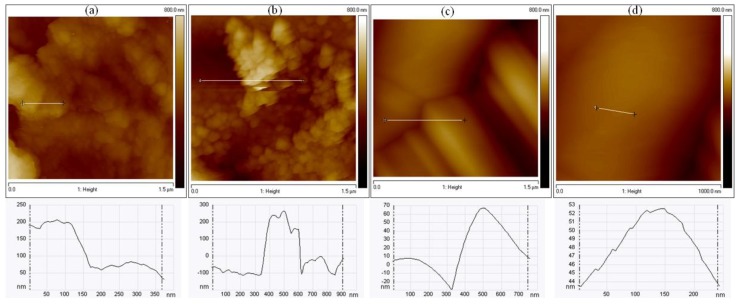
Atomic force microscopy images of samples C1 (**a**); C3 (**b**); and C2 (**c**,**d**). White line = profile traced to check the stacking of islands; result is shown in the chart below each image.

Figure 7 shows the surface morphology of the samples C1, C2 and C3. For samples C1 and C3 (Figure 7a,b), it is only possible to observe small isolated graphene islands. On the other hand, for sample C2 (Figure 7c,d), the graphene layers are larger and well distributed across the surface area compared to C1 and C3. The growth of the islands can be visualized in the profile of Figure 7d (sample C2), where stacking of the multilayers can be easily perceived. This result indicates that even a small difference in the energy density of the CO_2_ laser has a strong influence on the quality of the graphene grown on SiC film, which allows for the control of up to three orders of magnitude of material resistivity.

## 4. Conclusions

For the first time, we approached a feasible route for graphene growth based on the thermal decomposition of polycrystalline SiC thin films deposited by HiPIMS on AlN/Si substrates. For this purpose, we used CO_2_ laser beam heating without vacuum or controlled atmosphere. Raman mapping, along with AFM measurements, revealed the formation of islands of graphene on the SiC surfaces. The quality and density of the graphene islands was shown to be strongly dependent on the energy density of the laser process. It was observed that an energy density of the order of 137 J cm^−2^ allowed for obtaining interconnected graphene layers with some defects along the surface, resulting in a sheet resistance characteristic of graphene grown on pure crystal SiC wafer. Conversely, when the energy density was increased to 145 J cm^−2^, the density of the defects was considerably reduced. Finally, the results of this work demonstrate the feasibility of using the laser beam technique as a heating source for graphene formation on SiC thin films. This gives rise to new possibilities to explore the development of graphene layers on different substrates using the same methodology proposed here, which is a desirable situation for making different microelectronic and microelectromechanical systems (MEMS) devices. Given that the microfabrication processes are already well established for silicon wafers, there are still open issues related to SiC bulk etching.

## Figures and Tables

**Table 1:** Scanning velocities and energy densities applied for each condition.

	Condition 1 (C1)	Condition 2 (C2)	Condition 3 (C3)
**Scanning velocity (mm s^−1^)**	2300	2500	2600
**Energy density (J cm^−2^)**	145.25	136.95	127.69

**Table 2:** Sheet resistance of SiC thin film and graphene inferred by the four points probe method.

Sample	C1	C2	C3	SiC ^1^
Sheet resistance (Ω/□)	30,900	26	29,320	60,000

^1^ Reference measurement made on SiC film substrate.
